# Prophylactic Treatment of Undernourished Mice with Cotrimoxazole Induces a Different Profile of Dysbiosis with Functional Metabolic Alterations

**DOI:** 10.3390/cells11152278

**Published:** 2022-07-23

**Authors:** Lívia Budziarek Eslabão, Gabriela Farias Gubert, Lucas Cafferati Beltrame, Isis M. A. Mello, Oscar Bruna-Romero, Carlos R. Zárate-Bladés

**Affiliations:** 1 Laboratório de Imunorregulação, iREG, Departamento de Microbiologia, Imunologia e Parasitologia, Universidade Federal de Santa Catarina, Campus Universitário da Trindade, Florianópolis 88034-040, SC, Brazil; liviaeslabao@gmail.com (L.B.E.); gubert.gabriela@gmail.com (G.F.G.); lucasbeltrame97@gmail.com (L.C.B.); aisismello@gmail.com (I.M.A.M.); 2 Laboratório de Imunologia Aplicada, Departamento de Microbiologia, Imunologia e Parasitologia, Universidade Federal de Santa Catarina, Campus Universitário da Trindade, Florianópolis 88034-040, SC, Brazil

**Keywords:** microbiome, undernutrition, dysbiosis, cotrimoxazole, metabolism

## Abstract

Childhood malnutrition affects physiology and development. It increases infection rates, which may not present clinical signs in severe cases. The World Health Organization recommends prophylactic treatment with cotrimoxazole (SXT) and nutritional recovery to overcome this issue. This treatment is controversial, since evidence of a reduction in morbidity and mortality is not a consensus and could induce the development of antibiotic-resistant bacteria. Moreover, the impact of using this wide-spectrum antibiotic on gut microbiota in a critical period of development, and weakness is unknown. To understand how SXT prophylaxis could affect gut microbiota in undernutrition, we induced protein–energy undernutrition (PEU) in weaning C57BL/6 mice for three weeks and treated animals with SXT for two weeks. Using 16S rRNA gene sequencing, we compared the taxonomic composition and metabolic pathways of control mice, animals submitted to undernutrition (UND), treated with SXT, or undernourished and SXT treated (UND + SXT). We identified that UND mice had a significant increase in predicted pathways related to metabolic syndromes later in life. The prophylactic SXT treatment alone resulted in a significant loss in community richness and beta diversity. Furthermore, we identified the reduction of three genera in SXT treated mice, including the butyrate producers *Faecalibacterium* and *Anaerotruncus*. Both UND and double challenge (UND + SXT) resulted in a reduction of the amino acid’s biosynthesis pathway related to cell growth. Our results show that the SXT prophylaxis of young mice during an undernourishment period did not re-establish the undernourished microbiota community composition similar to healthy controls but induced a distinct dysbiotic profile with functional metabolic consequences.

## 1. Introduction

Undernutrition still affects approximately 200 million children every year. It is considered one of the leading underlying causes of morbidity and mortality, according to the World Health Organization (WHO) [1]. Undernutrition is defined as the imbalance between the nutrients and/or energy ingestion and the individual’s basic needs to sustain the body’s homeostasis and its specific functions and, in the case of infants, adequate growth [1]. Additionally, the acute form of malnutrition during childhood affects several organs and functions, from bones to neuronal development, metabolism, immunity, and even the gut microbiota [2,3,4].

In recent years, the microbiota has emerged as one of the major contributors to maintaining the individual’s health status, performing a range of functions from nutrients metabolism, development and modulation of the immune system, direct protection against infections, and even influencing behavior and cognition [5,6,7,8]. Several factors can greatly modulate the early gut microbiota during neonatal life, including mode of delivery, breastfeeding, use of antibiotics, environmental exposure, and nutritional status [9]. Understanding how nutrition and the gut microbiota of an individual interact with each other is essential to better comprehend the pathogenesis of undernutrition and develop better prevention measures and more effective treatments [10].

Children suffering from malnutrition are more prone to infection, but they may not show signs of clinical infection [11]. As a result, the WHO recommends a course of cotrimoxazole, a broad-spectrum antibiotic, as a prophylactic treatment for severely malnourished children [12]. The treatment is controversial, being classified by the WHO based on weak evidence [13]. Researchers found no increase in survival in a multicenter, double-blind, randomized controlled trial [14]. There is also the issue of global concern in increasing microbial resistance [15] and the potentiality of disruption of gut microbiota, resulting in broad-spectrum antibiotics-induced dysbiosis [16]. Nonetheless, there is also the opinion that the use of cotrimoxazole during undernutrition treatment could preserve the structure of the microbiome or even be beneficial for it, since it would avoid the invasion of pathogens during a period of particular vulnerability [17].

In this study, we reproduced human infant undernutrition and its treatment in mice. The aim was to define the effects of undernutrition, cotrimoxazole, and their association on microbiome composition and functionality. Our results show that each situation results in a different type of dysbiosis, and that the WHO’s malnutrition preconized treatment might not lead to the re-establishment of a healthy gut microbiome.

## 2. Materials and Methods

### 2.1. Animal and Study Design

C57BL/6 mice with the age of 3–4 weeks were randomly housed in groups of three animals per microisolator cage (Alesco, Campinas, Brazil) on sterilized wood chip bedding under a controlled temperature (21 ± 1 °C) and humidity (50 ± 20%) with a 12 h light/dark cycle. All animals were acclimated for four days to recover from transport stress before beginning the experimental protocol. The mice had ad libitum access to sterilized distilled water and an irradiated diet during the acclimation period. 

After acclimation, mice weighing 10.9 ± 1.52 g (mean ± SD) were randomly grouped into either the ad libitum control group or the undernourished group. The control group and the undernourished group were divided into two sub-groups to study the effect of antibiotic therapy on food intake and gut microbiota: no additional treatment and cotrimoxazole-treatment. Thus, the experiment was carried out in four groups: control group (CON) (n = 6), cotrimoxazole-treated group (SXT) (n = 6), undernourished group (UND) (n = 6), and undernourished and cotrimoxazole-treated group (UND + SXT) (n = 6). The undernourishment model protocol was adapted from Mittal and Woodward [18]. The undernourished groups (UND and UND + SXT) were fed with diets containing only 60% of the total food consumed by the CON and SXT groups, consisting of 1.4 g of food per animal consumed daily by the undernourished groups. Feeding was performed in the afternoon to respect the mice’s circadian rhythms. The food restriction protocol was sustained for three weeks. 

The SXT protocol was carried out for two weeks before the end of the food-restriction period. The mice in the SXT and UND + SXT groups were treated daily with SXT (a combination of 25 mg/kg of sulfamethoxazole + 5 mg/kg of trimethoprim) for two weeks, as recommended by the WHO [12] for cases of severe malnutrition without apparent infection. SXT was purchased from a veterinary pharmacy with banana flavoring for greater palatability to the animals. The drug application volume was adjusted to 50 μL, containing 10 μL of cotrimoxazole and 40 μL of ultrapure water (Merck KGaA, Darmstadt, Germany). Finally, the drug was administered orally through a cannula and syringe system. 

All procedures were performed following the Health Guide for the Care and Use of Laboratory Animals from the Brazilian College of Animal Experimentation and were approved by the Institution’s Ethics Committee under protocol number 5560250219. All efforts were made to minimize animal suffering and reduce the number of animals used in the experiments. 

### 2.2. Fecal Sampling and DNA Extraction

Fecal samples were collected individually from the mice at day 0 (before the food restriction protocol) and at day 21 (end of the food restriction protocol). Samples were collected from each mouse independently by performing a tail-lift and aseptically collecting the fecal content directly from the anus into sterile tubes. The fecal samples were immediately transferred to liquid nitrogen and subsequently stored at −80 °C until processing. 

According to the manufacturer’s recommendation, DNA extraction was performed on weighted fecal samples using a FastDNA™ SPIN Kit (MP Biomedicals, Santa Ana, CA, USA).

### 2.3. 16S rRNA Gene Sequence and Analysis

The DNA extracts were quantified with a Qubit dsDNA BR Assay Kit (Invitrogen™, Thermo Fisher Scientific, Carlsbad, CA, USA) and amplified using primers 341F (5′-CCTAYGGGRBGCASCAG-3′) and 806R (5′-GGACTACNNGGGTATCTAAT-3′) for the V3–V4 hypervariable region of the 16S rRNA gene associated with a barcode sequence, as described by Yu et al. [19]. Polymerase chain reactions were performed with 15 μL Phusion^®^ High-Fidelity PCR Master Mix (New England Biolabs, Ipswich, UK), 0.2 μM of forwarding primer, 0.2 μM of reverse primer, and 10 ng of template DNA. Amplification conditions consisted of an initial denaturation at 98 °C for 1 min, followed by 30 cycles of denaturation at 98 °C for 10 s, annealing at 50 °C for 10 s, and extension 72 °C for 60 s, with a final extension of 72 °C for 5 min. Amplification was confirmed through electrophoresis in agarose gel, resulting in amplicons with approximately 400–450 bp. Amplicons were purified with Qiagen Gel Extraction Kit (Qiagen, Hilden, Germany) and prepared using a TruSeq^®^ DNA PCR-Free Sample Preparation Kit (Illumina, San Diego, CA, USA) following the manufacturer’s instruction. Finally, the library was sequenced on an Illumina HiSeq 2500 (Illumina, San Diego, CA, USA), resulting in 250 bp paired-end reads. 16S rRNA gene sequence was performed by GenOne Biotech (Rio de Janeiro, Brazil). 

According to sequence size and Phred score, paired-end 16S rRNA gene sequences of low quality were filtered with Trimmomatic v0.36 [20]. Nucleotides with a Phred score under 33 at the beginning and end of each sequence and sequences shorter than 200 nucleotides were considered low quality and removed. Barcode and primer sequences were also removed. Paired-end sequences were merged using DADA2 [21], available with the Quantitative Insights Into Microbial Ecology 2 (QIIME 2) software [22]. Chimera removal, singletons filtering, amplicon sequence variant generation (ASV), and rare ASV removal were also assessed using DADA2 pipeline. The taxonomy was assigned according to VSEARCH using the Greengenes v13.8 database [23,24]. PICRUSt2 was used to predict metagenomic functions based on the normalized ASV tables [25].

### 2.4. Statistical Analysis of 16S rRNA Sequencing Data

All statistical analyses for 16S rRNA sequence data were performed in R v4.1.0 [26]. An alpha diversity measure of bacterial richness (observed species and Chao1) and diversity (Shannon and Simpson) were analyzed using the phyloseq R package [27]. The Student’s t-test or ANOVA followed by Tukey post hoc test were applied for parametric data, and a Mann–Whitney U test or Kruskal–Wallis followed by Dunn’s test of multiple comparisons for non-parametric data were applied to test the statistical significance of alpha diversity. The Bray–Curtis distance metrics were used to access beta diversity through the phyloseq R package [27] and vegan R package [28]. Multivariate permutation analysis of variance (ADONIS) was conducted with 10,000 permutations [29] to access the beta diversity statistical significance. A Principal Coordinates Analysis (PCoA) of the Bray–Curtis distance was performed using the vegan R package [28]. Differences in bacterial taxa abundance between experimental groups were evaluated using the Analysis of Compositions of Microbiomes with Bias Correction (ANCOM-BC) R package [30]. The ANCOM-BC methodology models the observed abundance through an offset-based log-linear regression model with a Benjamini–Hochberg False Discovery Rate (FDR) correction. Prediction metagenomic functions were analyzed through LEfSe analysis under the following conditions: the α value for the factorial Kruskal–Wallis test and pairwise Wilcoxon test among classes was <0.05 and the threshold on the logarithmic LDA score for discriminative features was >2.0. Significant data from LEfSe analysis were submitted to FDR correction for adjusted *p*-value. Data with *p* FDR < 0.05 were considered to be significant.

## 3. Results

### 3.1. Changes in Body Weight and Daily Weight Gain in C57BL/6 Mice during Undernourishment Induction

**Figure 1:**
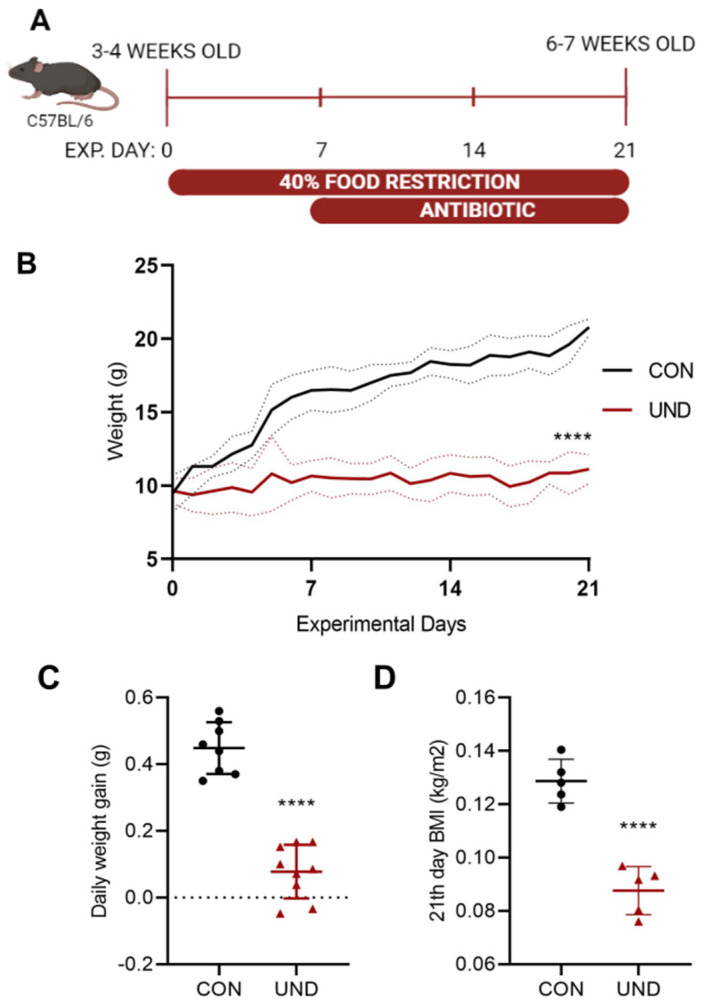
Changes in body weight during undernourishment induction through food restriction. (**A**) Experimental design, C57BL/6 mice with 3-4 weeks old were submitted to 40% food restriction for 21 experimental days (exp. day 21). (**B**) Body weight (g) changes in male C57BL/6 mice during the experimental protocol. (**C**) Daily weight gain (g) for the 3 weeks food restriction period. (**D**) BMI at the 21st experimental day. n = 5 animals per group. Each dot or triangle corresponds to a single mouse. Experiments were repeated at least three times. Data for one typical experiment are shown. Statistical significances were assessed by Mann-Whitney U test. Data expressed as mean ± SD. **** *p* < 0.0001.

C57BL/6 mice aged 3–4 weeks were randomly assigned to the CON or UND groups (Day –3). The mice from the CON group were offered food ad libitum. The undernourishment protocol consisted of a reduction of 40% of the daily food consumption for three weeks (from experimental day 0 to day 21) [18] (Figure 1A). 

The undernourishment protocol resulted in a significant body-weight loss compared to same-age healthy C57BL/6 mice (Figure 1B). The lowest body weight was observed at the end of the food-restriction protocol with undernourished mice weighing 10.99 g (± 1.18) and control mice weighing 19.62 g (±1.28) (*p* < 0.0001). As expected, the daily weight gain in undernourished mice (0.088 g ± 0.080) was significantly lower compared to the daily weight gain in control mice (0.449 g ± 0.078) (*p* < 0.0001) during the establishment of the food-restriction protocol (Figure 1C). At experimental day 21, the end of food restriction, the undernourished mice had a significantly lower body mass index (BMI) (0.088 kg/m^2^ ± 0.009) compared to the control mice (0.129 kg/m^2^ ± 0.008) (*p* < 0.0001).

### 3.2. Effects of Undernourishment on the Microbiota Composition and Function

**Figure 2:**
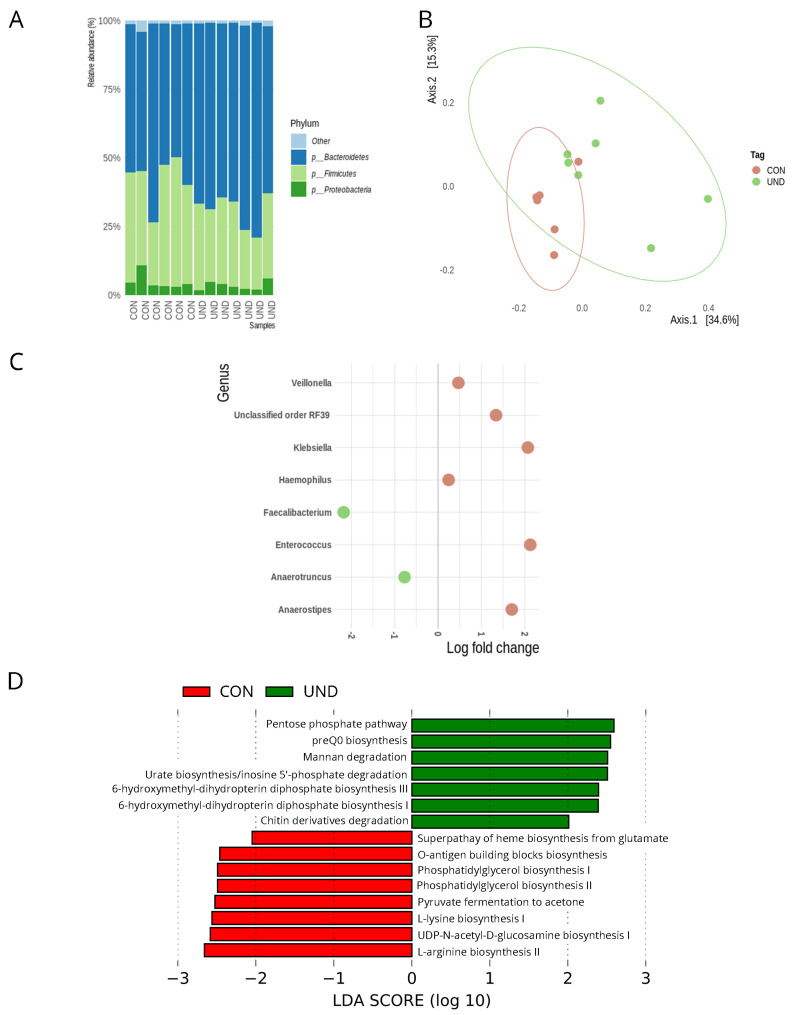
The gut microbiota composition and inferred functional content of gut microbiota between of control (CON) and undernourished (UND) C57BL/6 mice. (**A**) Relative abundance at the phylum level for groups of CON (n = 6) and UND (n = 7) C57BL/6 mice; (**B**) Principal coordinate analysis (PCoA) of Bray–Curtis distances among groups of CON (n = 6) and UND (n = 7) C57BL/6 mice. Each point corresponds to a community from a single mouse. Colors indicate group identity. Ellipses show the 95% confidence intervals. Intra-group differences were evaluated using ADONIS test (*p* < 0.05); (**C**) ANCOM-BC log fold change showing differentially abundant taxonomic clades in the gut microbiota of UND (n = 7) versus CON (n = 6) C57BL/6 mice; (**D**) LEfSe LDA scores showing significant pathway differences between CON (n = 6) and UND (n = 7) C57BL/6 mice of PICRUSt predicted the relative MetaCyc pathways abundances. Significant differences between groups were corrected with Benjamini–Hochberg False Discovery Rate *p* FDR < 0.05.

The gut microbiota was analyzed through 16S rRNA gene sequencing of fecal samples to study the effects of undernourishment on the microbiota composition and function. After data processing and quality checking, there were 2,879,810 reads with an average of 115,192 reads per sample. Sequences were clustered into ASVs, resulting in 11,126 ASVs with an average of 445 ASVs per sample. We analyzed the fecal microbiota composition between the CON and UND mice at the end of the food restriction period. Regarding the microbiota composition, 17 main phyla were found, of which Bacteroidetes, Firmicutes, and Proteobacteria were the most abundant (Figure 2A). Alpha diversity analysis showed no difference in community richness (Observed ASV and Chao1) and diversity indices (Shannon and Simpson) between CON and UND mice (Supplemental Figure S1). PCoA was performed using Bray–Curtis distances to illustrate the intra-group microbial community’s differences after the undernourishment protocol, revealing distinct findings (Figure 2B). The UND microbiota was significantly different in community composition when compared to the CON microbiota (ADONIS with 10,000 permutations, *p* = 0.005, *p* FDR = 0.005).

An ANCOM-BC analysis was used to compare the gut microbiota taxa that were significantly different between the CON and UND groups (Figure 2C). When comparing taxa at the genus level, UND mice presented an increase in *Veillonella* (*p* FDR < 0.05), unclassified genus from order RF39 (*p* FDR < 0.05), *Klebsiella* (*p* FDR < 0.05), *Haemophilus* (*p* FDR < 0.05), *Enterococcus* (*p* FDR < 0.05), and *Anaerostipes* (*p* FDR < 0.05). Additionally, UND mice showed a decrease in *Faecalibacterium* (*p* FDR < 0.05) and *Anaerotruncus* (*p* FDR < 0.05).

Metabolic pathways were accessed using PICRUSt2 to determine whether the observed taxonomic differences between groups played a role in their functions. LEfSe analysis compared metabolic changes in the gut microbiota in each group (Figure 2D). All displayed pathways presented an LDA score > 2.0. The UND group changes were mainly related to cellular growth. Pathways related to the biosynthesis of sugar nucleotides [O-antigen building blocks biosynthesis (0.066 ± 0.004 vs. 0.073 ± 0.005; *p* < 0.001; *p* FDR < 0.001), UDP-N-acetyl-D-glucosamine biosynthesis I (0.052 ± 0.006 vs. 0.062 ± 0.007; *p* < 0.001; *p* FDR < 0.001)], amino acids [L-lysine biosynthesis I (0.074 ± 0.006 vs. 0.083 ± 0.006; *p* < 0.001; *p* FDR < 0.001), L-arginine biosynthesis II (0.081 ± 0.009 vs. 0.092 ± 0.006; *p* < 0.001; *p* FDR < 0.001)], cell membrane lipids [phosphatidylglycerol biosynthesis I (0.0921 ± 0.003 vs. 0.099 ± 0.006; *p* < 0.001; *p* FDR < 0.001), and phosphatidylglycerol biosynthesis II (0.0921 ± 0.003 vs. 0.099 ± 0.006; *p* < 0.001; *p* FDR < 0.001)] were less present compared to the CON group. Moreover, with the compromising of growth-related metabolic pathways, UND presented increases in cell component degradation pathways, such as carbohydrates [pentose phosphate pathway (0.073 ± 0.008 vs. 0.062 ± 0.005; *p* < 0.001; *p* FDR < 0.001)], polysaccharides [mannan degradation (0.047 ± 0.007 vs. 0.038 ± 0.005; *p* < 0.001; *p* FDR < 0.001)], purine nucleotides [urate biosynthesis/inosine 5’-phosphate degradation (0.112 ± 0.005 vs. 0.101 ± 0.008; *p* < 0.001; *p* FDR < 0.001)], and cellular wall [chitin derivatives degradation (0.00011 ± 0.00022 vs. 0 ± 0; *p* < 0.001; *p* FDR < 0.001)]. There was also an increase in the vitamin biosynthesis-related pathways of preQ0 (0.067 ± 0.007 vs. 0.05 ± 0.006; *p* < 0.001; *p* FDR < 0.001), 6-hydroxymethyl-dihydropterin diphosphate III (0.097 ± 0.005 vs. 0.088 ± 0.007; *p* < 0.001; *p* FDR < 0.001), and 6-hydroxymethyl-dihydropterin diphosphate I (0.096 ± 0.005 vs. 0.087 ± 0.007; *p* < 0.001; *p* FDR < 0.001).

### 3.3. Effects of Cotrimoxazole (SXT) on the Gut Microbiome

**Figure 3:**
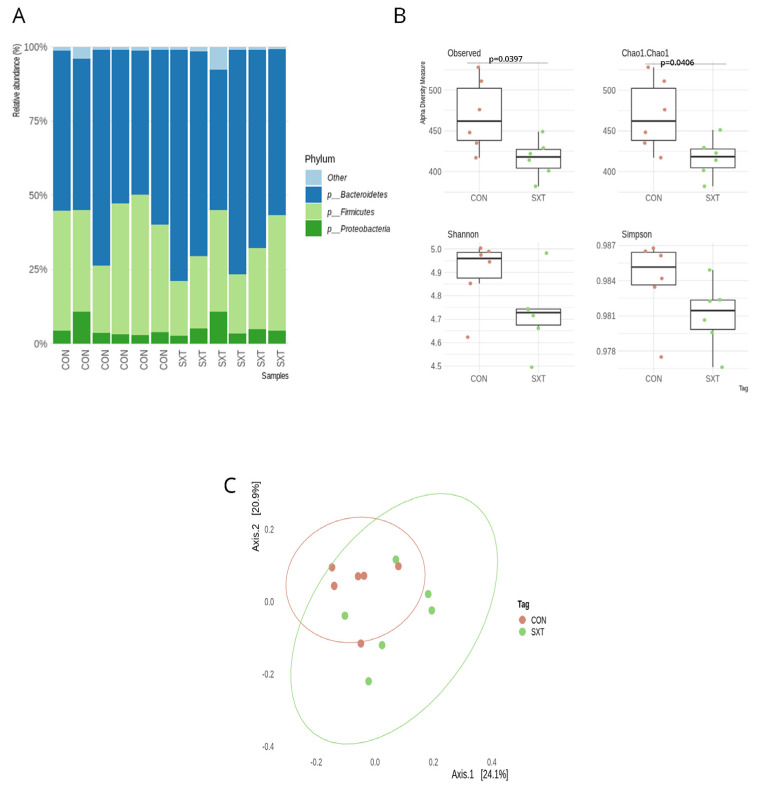
The gut microbiota composition of gut microbiota between of control (CON) and cotrimoxazole (SXT) C57BL/6 mice. (**A**) Relative abundance at the phylum level for groups of CON (n = 6) and SXT (n = 6) C57BL/6 mice; (**B**) richness (Observed and Chao1) and diversity (Shannon and Simpson) indexes for groups of CON (n = 6) and SXT (n = 6) C57BL/6 mice; (**C**) PCoA of Bray-Curtis distances among groups of CON (n = 6) and SXT (n = 6) C57BL/6 mice. Each point corresponds to a community from a single mouse. Colors indicate group identity. Ellipses show the 95% confidence intervals. Intra-group differences were evaluated using ADONIS test (*p* < 0.05).

We analyzed the fecal microbiota composition between the mice from the CON and SXT groups at the end of the antibiotic treatment to study the effect of the SXT treatment on the microbiota compositions and functions. Regarding the microbiota composition, the same 17 main phyla found in UND were present in SXT (Figure 3A). An alpha diversity analysis showed a significant difference in community richness (Observed, *p* = 0.032, *p* FDR = 0.03204; and Chao1, *p* = 0.033, *p* FDR = 0.0336) between CON and SXT mice (Figure 3B). In contrast, there was no difference in diversity indices (Shannon and Simpson) between the groups (Figure 3B). The PCoA was performed using Bray–Curtis distances to illustrate microbial community changes after the antibiotic treatment (Figure 3C). The SXT microbiota was significantly different regarding community composition following the antibiotic treatment compared to the CON microbiota (ADONIS, *p* = 0.021, *p* FDR = 0.0218).

**Figure 4:**
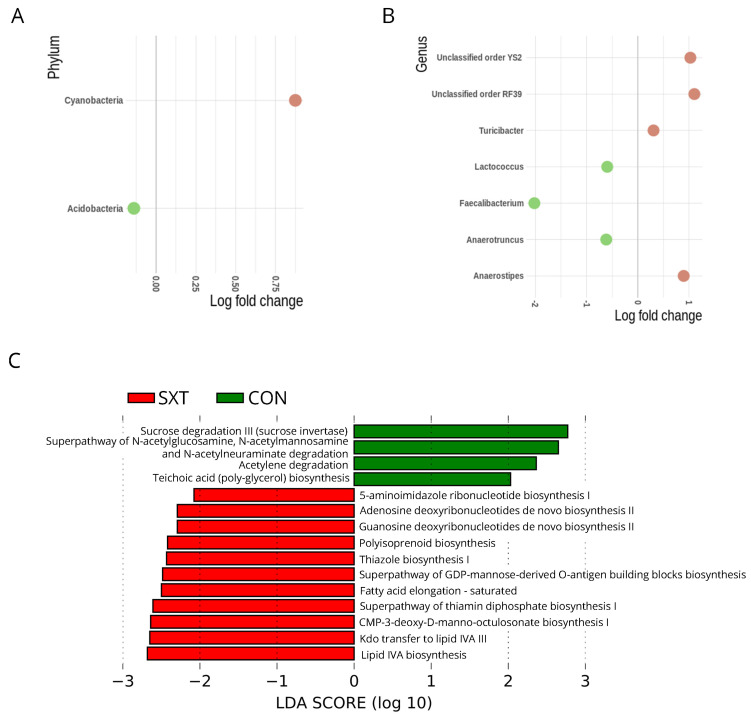
The gut microbiota composition and inferred functional content of gut microbiota between control (CON) and cotrimoxazole (SXT) C57BL/6 mice. (**A**) ANCOM-BC log fold change showing differentially abundant taxonomic phylum in the gut microbiota of SXT (n = 6) versus CON (n = 6) C57BL/6 mice; (**B**) ANCOM-BC log fold change showing differentially abundant taxonomic genus in the gut microbiota of SXT (n = 6) versus CON (n = 6) C57BL/6 mice; (**C**) LEfSe LDA scores showing significant pathway differences between CON (n = 6) and SXT (n = 6) C57BL/6 mice of PICRUSt predicted the relative MetaCyc pathways abundances. Significant differences between groups were corrected with Benjamini–Hochberg False Discovery Rate *p* FDR < 0.05.

ANCOM-BC analysis was used to compare the gut microbiota taxa that were significantly different between the CON and SXT groups (Figure 4A,B). We identified differences at a phylum level; SXT mice presented an increase in Cyanobacteria and a decrease in Acidobacteria (FDR < 0.05) (Figure 4A). We also observed differences at a genus level; SXT mice had an increase in *Anaerostipes*, *Turicibacter* and unclassified genera from order YS2 and order RF39 (FDR < 0.05) (Figure 4B). In addition to that, SXT mice showed a decrease in *Lactococcus*, *Faecalibacterium*, and *Anaerotruncus* genera (FDR < 0.05) (Figure 4B).

Figure 4C showed the LEfSe analysis comparing metabolic changes in the gut microbiota of CON and SXT mice. SXT increased pathways related to nucleotide synthesis [5-aminoimidazole ribonucleotide biosynthesis I (0.113 ± 0.003 vs. 0.1104 ± 0.003; *p* < 0.001; *p* FDR < 0.001); adenosine deoxyribonucleotides de novo biosynthesis II (0.09398 ± 0.002 vs. 0.093 79 ± 0.002; *p* < 0.001; *p* FDR < 0.001); guanosine deoxyribonucleotides de novo biosynthesis II (0.09398 ± 0.002 vs. 0.093 79 ± 0.002; *p* < 0.001; *p* FDR < 0.001); superpathway of GDP-mannose-derived O-antigen building blocks biosynthesis (0.072 ± 0.003 vs. 0.068 ± 0.003; *p* < 0.001; *p* FDR < 0.001); CMP-3-deoxy-D-manno-octulosonate biosynthesis I (0.0627 ± 0.003 vs. 0.0553 ± 0.005; *p* < 0.001; *p* FDR < 0.001)], vitamin synthesis [thiazole biosynthesis I (0.0501 ± 0.003 vs. 0.0457 ± 0.004; *p* < 0.001; *p* FDR < 0.001); superpathway of thiamin diphosphate biosynthesis I (0.0803 ± 0.003 vs. 0.0743 ± 0.004; *p* < 0.001; *p* FDR < 0.001)], lipid metabolism [fatty acid elongation (0.098 ± 0.001 vs. 0.095 ± 0.002; *p* < 0.001; *p* FDR < 0.001)], and alcohols [polyisoprenoid biosynthesis (0.083 ± 0.001 vs. 0.08 ± 0.001; *p* < 0.001; *p* FDR < 0.001)]. In contrast, SXT decreased the carbohydrate and amine degradation [sucrose degradation III (0.055 ± 0.006 vs. 0.0724 ± 0.012; *p* < 0.001; *p* FDR < 0.001); superpathway of N-acetylglucosamine, N-acetylmannosamine and N-acetylneuraminate degradation (0.0403 ± 0.003 vs. 0.0536 ± 0.008; *p* < 0.001; *p* FDR < 0.001)], fermentation of pyruvate or short-chain fatty acids [acetylene degradation (0.0244 ± 0.004 vs. 0.031 ± 0.004; *p* < 0.001; *p* FDR < 0.001)], and formation of cell wall [teichoic acid biosynthesis (0.006 ± 0.001 vs. 0.009 ± 0.001; *p* < 0.001; *p* FDR < 0.001)] compared to CON mice.

### 3.4. The Use of Cotrimoxazole during Undernourishment in Infant Mice Does Not Revert Undernutrition Effects on Microbiota but Results in a Distinct Profile of Dysbiosis

**Figure 5:**
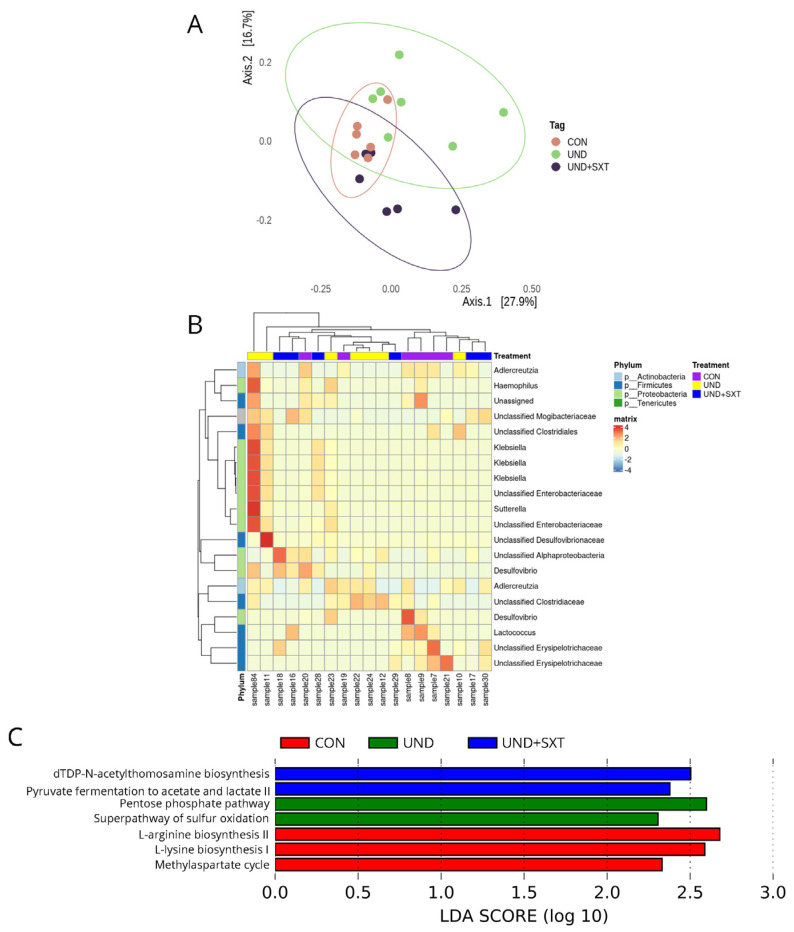
The gut microbiota composition and inferred functional content of gut microbiota between of control (CON), undernourished (UND), and cotrimoxazole-treated undernourished (UND-SXT) C57BL/6 mice. (**A**) PCoA of Bray–Curtis distances among groups of CON (n = 6), UND (n = 7), and SXT (n = 6) C57BL/6 mice. Each point corresponds to a community from a single mouse. Colors indicate group identity. Ellipses show the 95% confidence intervals. Intra-group differences were evaluated using ADONIS test (*p* < 0.05); (**B**) heatmap representation of ANCOM-BC analysis showing abundance for the 20 most differently significant genus (*p* < 0.0001) in the gut microbiota of CON (n = 6), UND (n = 7), and SXT (n = 6) C57BL/6 mice; (**C**) LEfSe LDA scores showing significant pathway differences between CON (n = 6), UND (n = 7), and UND + SXT (n = 6) C57BL/6 mice of PICRUSt predicted the relative MetaCyc pathways abundances. Significant differences between groups were tested with Kruskal–Wallis test (*p* < 0.05).

The undernutrition treated with a prophylactic course of cotrimoxazole did not alter alpha diversity compared to UND and CON (Supplemental Figure S1). However, the CON, UND, and UND + SXT microbiota profiles are significantly different in community composition compared to each other (ADONIS, *p* = 0.0001, *p* FDR = 0.00019) (Figure 5A) (Supplemental Table S1). 

A heatmap representation of the ANCOM-BC analysis showed that UND had a significant increase in most of the microbiome composition when compared to the CON and UND + SXT mice (Figure 5B) (Supplemental Table S1). We observed an increase in *Haemophilus*, unclassified Clostridiales, *Klebsiella*, unclassified Enterobacteriaceae, *Sutterella*, unclassified Desulfovibrionaceae, *Adlercreutzia*, and unclassified Clostridiaceae. Whereas UND+SXT presented significant increase of *Anaerotruncus* genus and the decrease of *Sutterella, Parabacteroides, Lactococcus, Haemophilus, Candidatus Arthromitus, Streptococcus, Veillonella, Odoribacter, Anaerostipes, Adlercreutzia and Clostridium* genus (Supplemental Table S1). 

The LEfSe analysis comparing the metabolic changes in the gut microbiota of the control mice, undernourished mice, and double-treatment mice are presented in Figure 5C. The microbiota of UND + SXT mice presented a reduction in pathways related to energy production from organic substrates [methylaspartate cycle (0.00001 ± 0.00001 vs. 0.00006 ± 0.00006 vs. 0.00009 ± 0.00005 for UND + SXT, UND, and CON, respectively; *p* < 0.001; *p* FDR < 0.001)] and inorganic nutrient metabolism [superpathway of sulfur oxidation (0.02 ± 0.002 vs. 0.034 ± 0.012 vs. 0.027 ± 0.0064 for UND + SXT, UND, and CON, respectively; *p* < 0.001; *p* FDR < 0.001)] when compared to the CON and UND mice. The UND + SXT microbiota also presented an increase in pathways related to sugar nucleotide biosynthesis [dTDP-N-acetylthomosamine biosynthesis (0.071 ± 0.008 vs. 0.052 ± 0.011 vs. 0.065 ± 0.017 for UND + SXT, UND, and CON, respectively; *p* < 0.001; *p* FDR < 0.001)] and short-chain fatty acids fermentation [pyruvate fermentation to acetate and lactate II (0.332 ± 0.005 vs. 0.329 ± 0.012 vs. 0.325 ± 0.009 for UND + SXT, UND, and CON, respectively; *p* < 0.001; *p* FDR < 0.001)]. Moreover, UND microbiota presented an increase in the generation of precursor metabolites and energy pathways [pentose phosphate pathway (0.155 ± 0.024 vs. 0.187 ± 0.027 vs. 0.152 ± 0.018 for UND + SXT, UND, and CON, respectively; *p* < 0.001; *p* FDR < 0.001)]. Furthermore, CON microbiota presented an increase in pathways related to amino acid biosynthesis [L-lysine biosynthesis I (0.196 ± 0.009 vs. 0.189 ± 0.011 vs. 0.202 ± 0.012 for UND + SXT, UND, and CON, respectively; *p* < 0.001; *p* FDR < 0.001) and L-arginine biosynthesis II (0.225 ± 0.014 vs. 0.207 ± 0.017 vs. 0.227 ± 0.013 for UND + SXT, UND, and CON, respectively; *p* < 0.001; *p* FDR < 0.001)].

## 4. Discussion

In the present study, we characterized the gut microbiota profiles of undernourished (UND), cotrimoxazole-treated (SXT), and undernourished cotrimoxazole-treated (UND + SXT) C57BL/6 mice after three weeks of protein–energy undernourishment and compared them to healthy control mice (CON). Our experimental protocol was carried out in animals just after weaning, intended to resemble undernutrition in children. Moreover, the main objective of our study was to establish the effects of SXT on the microbiota of undernourished mice, a wide-spectrum antibiotic therapy recommended by the WHO and routinely used in undernourished children.

First, our results show that C57BL/6 mice who underwent three weeks of protein–energy undernourishment presented no significant differences in fecal microbiota richness compared to healthy controls. However, they did present a significant change in beta diversity analysis, findings which were also observed by Dinh et al. [31]. We did not observe major phylum-level alterations in UND mice compared to CON mice. We reported an increase in *Veillonella*, *Klebsiella*, *Haemophilus*, *Enterococcus*, and *Anaerostipes*, all consisting of anaerobic or facultative anaerobic genera. These findings support clinical studies where the infants’ malnutrition was associated with reducing anaerobic bacteria [32,33,34]. The *Veillonella* genus was previously related to the nutrition status deterioration of Indian children [35]. Monira et al. found that the *Klebsiella* genus was 174-fold higher in malnourished children from Bangladesh [34]. Regarding the reduced genus *Faecalibacterium*, our data lines up with Subramanian et al.’s study [4], which found this genus depleted in children with severe acute malnutrition from Bangladesh, and Monira et al.’s [34] study, which correlated the presence of this genus with a better nutrition status in children. We also predicted differences in metabolic pathways based on microbiota composition. In UND mice, the pathways involved in the biosynthesis and/or the degradation of carbohydrates, polysaccharides, purine nucleotides, and the cellular wall were increased. Although not yet fully elucidated, evidence indicates that gut bacteria directly affect host urate degradation [36]. Our model predicted an increase in the purine nucleotides degradation pathway, which leads to the urate biosynthesis, suggesting a possible onset of hyperuricemia and probably leading to metabolic dysfunctions later in life. Moreover, UND mice increased the pentose phosphate pathway, a metabolic pathway involved in glucose oxidation. The pentose phosphate pathway is increased in the gut microbiome after depleting body glycogen stores during periods of insufficient carbohydrate consumption [37], which is compatible with carbohydrate consumption limitation during food-restriction ingestion. Our results should be considered an exploratory analysis to guide future investigations once we conducted a LEfSe analysis, a methodology that uses effect size to filter significant events rather than FDR correction [38]. 

Despite the efforts to achieve better clinical outcomes, 10 to 15% of undernourished children cannot recover even after controlled treatment [39]. Several studies have reported a higher prevalence of clinically significant infections among children who have been hospitalized for severe malnutrition [40,41]. However, diagnosing severe infection is difficult during undernutrition, since affected children might not present clinical signs of infection [40], hence the WHO’s recommendation for the SXT prophylactic treatment along with the ready-to-use therapeutic food (RUTF). Adding to the debate regarding the WHO’s guidelines, we first characterized the antibiotic-driven disruption of this wide-spectrum antibiotic in healthy infant mice. The SXT mice had a significant loss in community richness (Observed and Chao1) but no changes in diversity indices (Shannon and Simpson). In the case of HIV-exposed infants, SXT therapy did not change the microbiome taxonomic composition or functional metabolic pathways [42,43]. Other studies with HIV and hematological patients found no significant difference in α-diversity in the gut microbiome after SXT prophylaxis [43,44]. Nonetheless, according to beta diversity analysis, SXT treatment resulted in a microbiota community profile distinct from healthy control animals, with four bacterial genera enhanced and three genera reduced. Our data suggest that SXT treatment led to the reduction of *Faecalibacterium*, a genus with major involvement in amino acids [45] and SCFA [46] metabolism, maintenance of regulatory T cells immune response [47], and intestinal epithelial barrier integrity [48]. The SXT treatment also led to the reduction of *Lactococcus*, an abundant member of the family Streptococcaceae, usually associated with a beneficial impact on its host because of its ability to produce bacteriocins and organic acids [49]. Additionally, we observed a decrease in *Anaerotruncus*, a genus that consists of butyrate-producing bacteria, after SXT treatment [50]. However, the increase in *Anaerostipes*, a lactate-utilizing, butyrate-producing bacteria, might compensate for the reduction of other SCFA routes in the SXT mice [51]. Together, our results suggest that SXT prophylaxis favors a gut microenvironment with fewer taxa responsible for SCFA production and immune system stimulation in addition to impairing the colonization of opportunistic pathogens. 

The superpathway of GDP-mannose-derived O-antigen building blocks biosynthesis was elevated in the microbiome of SXT mice. The O-antigen is part of the bacteria’s outer lipopolysaccharide membrane [52]. Another molecule that constitutes the LPS is Lipid A [52]. The super pathway of (Kdo)2-lipid A biosynthesis was elevated in SXT mice, supporting the idea of a Gram-negative bacteria increase in STX-treated mice. LPS produced by healthy gut microbiota has an important role in the immunotolerance of the microbial community and was reduced in SXT mice [53]. 

Although gut microbiota modifications related to cotrimoxazole therapy are available in the literature, its effects in the undernourished gut microbiota and metabolic functions are still lacking. To elucidate the impact of SXT in the gut of undernourished mice, we compared undernourished mice treated with a prophylactic course of this antibiotic to healthy control mice. The microbiome taxonomic composition comparing the healthy control, UND, and UND + SXT mice demonstrated that the introduction of an SXT prophylactic course to undernourished mice did not fully recover the microbiota composition to a healthier profile. Compared to healthy controls, the reduction of the amino acid’s biosynthesis pathway in both UND and UND + SXT animals probably reflects the lack of food intake, which impacts cell growth. However, this lack of amino-acid synthesis could influence host physiology, as up to 20% of circulating plasma lysine, body protein lysine, and urinary lysine are derived from microbial sources [54]. In addition to being a precursor for protein biosynthesis [55], lysine regulates other amino acid synthesis pathways, such as arginine [56], which was also downregulated. Both lysine and arginine have been found to be reduced in stunted children [57]. 

Our study is the first to evaluate the effects of SXT prophylaxis on the undernourished gut microbiota of young animals. Nonetheless, it was not exempt from limitations, including experimental confirmation of functional predictions, evaluation of changes in the synthesis of specific gut bacteria-derived metabolites, and longer time of evaluation; these could inform the duration of the dysbiosis observed in each protocol and are points that should be addressed in future research projects. 

## 5. Conclusions

Altogether, our results present the impact of the prophylactic cotrimoxazole treatment on gut microbiota and the associated metabolic functional prediction in a murine model of childhood undernutrition. The controversial treatment alters gut microbiota differently from undernutrition or SXT alone, creating a third dysbiotic profile that alters the metabolic pathways related to amino acid synthesis and energy production. Additional studies are necessary to determine if the functionl predictions presented here affect enterocyte metabolism, intestine permeability, or taxonomic changes related to the host immune system.

## Data Availability

The data presented in this study are available on request from the corresponding author.

## Supplementary Materials

The following are available online at https://www.mdpi.com/article/10.3390/cells11152278/s1, Figure S1: Richness (Observed and Chao1) and diversity (Shannon and Simpson) indexes for the gut microbiota. (a) Control (CON) and undernourished (UND) C57BL/6 mice. (b) Control (CON) and cotrimoxazole-treated undernourished (UND-SXT) C57BL/6 mice. (c) Control (CON), undernourished (UND), and cotrimoxazole-treated undernourished (UND-SXT) C57BL/6 mice. Table S1: ANCOM-BC analysis showing W test statistic (mean difference/SE), *p*-value and FRD *p*-value for differently significant genus in the gut microbiota of CON (n = 6), UND (n = 7), and UND+SXT (n = 6) C57BL/6 mice. * Increased in UND+SXT mice when compared to CON and UND mice; ** reduced in UND+SXT mice when compared to CON and UND mice.

## References

[1] Hayashi C., Krasevec J., Kumapley R., United Nations Children’s Fund, World Health Organization, World Bank Group, International Bank for Reconstruction and Development (2021). Levels and Trends in Child Malnutrition: Key Findings of the 2021 Edition of the Joint Child Malnutrition Estimates.

[2] Giallourou N., Fardus-Reid F., Panic G., Veselkov K., McCormick B.J.J., Olortegui M.P., Ahmed T., Mduma E., Yori P.P., Mahfuz M. (2020). Metabolic maturation in the first 2 years of life in resource-constrained settings and its association with postnatal growths. Sci. Adv..

[3] Ibrahim M.K., Zambruni M., Melby C.L., Melby P.C. (2017). Impact of Childhood Malnutrition on Host Defense and Infection. Clin. Microbiol. Rev..

[4] Subramanian S., Huq S., Yatsunenko T., Haque R., Mahfuz M., Alam M.A., Benezra A., DeStefano J., Meier M.F., Muegge B.D. (2014). Persistent gut microbiota immaturity in malnourished Bangladeshi children. Nature.

[5] Lynn D.J., Pulendran B. (2017). The potential of the microbiota to influence vaccine responses. J. Leukoc. Biol..

[6] Chen Y., Zhou J., Wang L. (2021). Role and Mechanism of Gut Microbiota in Human Disease. Front. Cell. Infect. Microbiol..

[7] Jiao Y., Wu L., Huntington N.D., Zhang X. (2020). Crosstalk Between Gut Microbiota and Innate Immunity and Its Implication in Autoimmune Diseases. Front. Immunol..

[8] Tooley K.L. (2020). Effects of the human gut microbiota on cognitive performance, brain structure and function: A narrative review. Nutrients.

[9] Jeong S. (2021). Factors influencing development of the infant microbiota: From prenatal period to early infancy. Clin. Exp. Pediatr..

[10] Kau A.L., Planer J.D., Liu J., Rao S., Yatsunenko T., Trehan I., Manary M.J., Liu T.-C., Stappenbeck T.S., Maleta K.M. (2015). Functional characterization of IgA-targeted bacterial taxa from undernourished Malawian children that produce diet-dependent enteropathy. Sci. Transl. Med..

[11] Jones K.D.J., Berkley J.A. (2014). Severe acute malnutrition and infection. Paediatr. Int. Child Health.

[12] World Health Organization (1999). Management of Severe Malnutrition: A Manual for Physicians and Other Senior Health Workers.

[13] Williams P.C.M., Berkley J.A. (2016). Sever Acute Malnutrition Update: Current WHO Guidelines and the WHO Essential Medicine List for Children.

[14] A Berkley J., Ngari M., Thitiri J., Mwalekwa L., Timbwa M., Hamid F., Ali R., Shangala J., Mturi N., Jones K. (2016). Daily co-trimoxazole prophylaxis to prevent mortality in children with complicated severe acute malnutrition: A multicentre, double-blind, randomised placebo-controlled trial. Lancet Glob. Health.

[15] Laxminarayan R., Duse A., Wattal C., Zaidi A.K.M., Wertheim H.F.L., Sumpradit N., Vlieghe E., Hara G.L., Gould I.M., Goossens H. (2013). Antibiotic resistance—The need for global solutions. Lancet Infect. Dis..

[16] Ramirez J., Guarner F., Fernandez L.B., Maruy A., Sdepanian V.L., Cohen H. (2020). Antibiotics as Major Disruptors of Gut Microbiota. Front. Cell. Infect. Microbiol..

[17] Jones K.D., Thitiri J., Ngari M., Berkley J.A. (2014). Childhood malnutrition: Toward an understanding of infections, inflammation, and antimicrobials. Food Nutr. Bull..

[18] Mittal A., Woodward B. (1985). Thymic Epithelial Cells of Severely Undernourished Mice: Accumulation of Cholesteryl Esters and Absence of Cytoplasmic Vacuoles. Exp. Biol. Med..

[19] Yu Y., Lee C., Kim J., Hwang S. (2005). Group-specific primer and probe sets to detect methanogenic communities using quantitative real-time polymerase chain reaction. Biotechnol. Bioeng..

[20] Bolger A.M., Lohse M., Usadel B. (2014). Trimmomatic: A flexible trimmer for Illumina sequence data. Bioinformatics.

[21] Callahan B.J., Mcmurdie P.J., Rosen M.J., Han A.W., Johnson A.J.A., Holmes S.P. (2016). DADA_2_: High-resolution sample inference from Illumina amplicon data. Nat. Methods.

[22] Bolyen E., Rideout J.R., Dillon M.R., Bokulich N.A., Abnet C.C., Al-Ghalith G.A., Alexander H., Alm E.J., Arumugam M., Asnicar F. (2019). Reproducible, interactive, scalable and extensible microbiome data science using QIIME 2. Nat. Biotechnol..

[23] DeSantis T.Z., Hugenholtz P., Larsen N., Rojas M., Brodie E.L., Keller K., Huber T., Dalevi D., Hu P., Andersen G.L. (2006). Greengenes, a chimera-checked 16S rRNA gene database and workbench compatible with ARB. Appl. Environ. Microbiol..

[24] Rognes T., Flouri T., Nichols B., Quince C., Mahé F. (2016). VSEARCH: A versatile open source tool for metagenomics. PeerJ.

[25] Douglas G.M., Maffei V.J., Zaneveld J.R., Yurgel S.N., Brown J.R., Taylor C.M., Huttenhower C., Langille M.G.I. (2020). PICRUSt2 for prediction of metagenome functions. Nat. Biotechnol..

[26] R Core Team The R Project for Statistical Computing. R Foundation for Statistical Computing Web-Site. www.R-project.org.

[27] McMurdie P.J., Holmes S. (2013). phyloseq: An R Package for Reproducible Interactive Analysis and Graphics of Microbiome Census Data. PLoS ONE.

[28] Oksanen J., Blanchet F.G., Friendly M., Kindt R., Legendre P., McGlinn D., Minchin P.R., O’Hara R.B., Simpson G.L., Solymos P. Vegan: Community Ecology Package. https://cran.r-project.org/web/packages/vegan/index.html.

[29] Anderson M.J., Walsh D.C.I. (2013). PERMANOVA, ANOSIM, and the Mantel test in the face of heterogeneous dispersions: What null hypothesis are you testing?. Ecol. Monogr..

[30] Lin H., Das Peddada S. (2020). Analysis of compositions of microbiomes with bias correction. Nat. Commun..

[31] Dinh D.M., Ramadass B., Kattula D., Sarkar R., Braunstein P., Tai A., Wanke C.A., Hassoun S., Kane A.V., Naumova E.N. (2016). Longitudinal Analysis of the Intestinal Microbiota in Persistently Stunted Young Children in South India. PLoS ONE.

[32] Million M., Alou M.T., Khelaifia S., Bachar D., Lagier J.-C., Dione N., Brah S., Hugon P., Lombard V., Armougom F. (2016). Increased Gut Redox and Depletion of Anaerobic and Methanogenic Prokaryotes in Severe Acute Malnutrition. Sci. Rep..

[33] Smith M.I., Yatsunenko T., Manary M.J., Trehan I., Mkakosya R., Cheng J., Kau A.L., Rich S.S., Concannon P., Mychaleckyj J.C. (2013). Gut Microbiomes of Malawian Twin Pairs Discordant for Kwashiorkor. Science.

[34] Monira S., Nakamura S., Gotoh K., Izutsu K., Watanabe H., Alam N.H., Endtz H.P., Cravioto A., Ali S.I., Nakaya T. (2011). Gut Microbiota of Healthy and Malnourished Children in Bangladesh. Front. Microbiol..

[35] Ghosh T., Gupta S.S., Bhattacharya T., Yadav D., Barik A., Chowdhury A., Das B., Mande S.S., Nair G.B. (2014). Gut Microbiomes of Indian Children of Varying Nutritional Status. PLoS ONE.

[36] Chu Y., Sun S., Huang Y., Gao Q., Xie X., Wang P., Li J., Liang L., He X., Jiang Y. (2021). Metagenomic analysis revealed the potential role of gut microbiome in gout. npj Biofilms Microbiomes.

[37] Zhao X., Zhang Z., Hu B., Huang W., Yuan C., Zou L. (2018). Response of gut microbiota to metabolite changes induced by endurance exercise. Front. Microbiol..

[38] Nearing J.T., Douglas G.M., Hayes M.G., MacDonald J., Desai D.K., Allward N., Jones C.M.A., Wright R.J., Dhanani A.S., Comeau A.M. (2022). Microbiome differential abundance methods produce different results across 38 datasets. Nat. Commun..

[39] A Ciliberto M., Sandige H., Ndekha M.J., Ashorn P., Briend A., Ciliberto H.M., Manary M.J. (2005). Comparison of home-based therapy with ready-to-use therapeutic food with standard therapy in the treatment of malnourished Malawian children: A controlled, clinical effectiveness trial. Am. J. Clin. Nutr..

[40] Bahwere P., Levy J., Hennart P., Donnen P., Lomoyo W., Dramaix-Wilmet M., Hemelof W., Butzler J.-P., De Mol P. (2001). Community-acquired bacteremia among hospitalized children in rural central Africa. Int. J. Infect. Dis..

[41] Berkley J.A., Lowe B.S., Mwangi I., Williams T., Bauni E., Mwarumba S., Ngetsa C., Slack M.P., Njenga S., Hart C.A. (2005). Bacteremia among Children Admitted to a Rural Hospital in Kenya. N. Engl. J. Med..

[42] D’Souza A.W., Moodley-Govender E., Berla B., Kelkar T., Wang B., Sun X., Daniels B., Coutsoudis A., Trehan I., Dantas G. (2020). Cotrimoxazole Prophylaxis Increases Resistance Gene Prevalence and α-Diversity but Decreases β-Diversity in the Gut Microbiome of Human Immunodeficiency Virus–Exposed, Uninfected Infants. Clin. Infect. Dis..

[43] Bourke C.D., Gough E.K., Pimundu G., Shonhai A., Berejena C., Terry L., Baumard L., Choudhry N., Karmali Y., Bwakura-Dangarembizi M. (2019). Cotrimoxazole reduces systemic inflammation in HIV infection by altering the gut microbiome and immune activation. Sci. Transl. Med..

[44] Willmann M., Vehreschild M.J.G.T., Biehl L.M., Vogel W., Dörfel D., Hamprecht A., Seifert H., Autenrieth I.B., Peter S. (2019). Distinct impact of antibiotics on the gut microbiome and resistome: A longitudinal multicenter cohort study. BMC Biol..

[45] Roager H.M., Licht T.R. (2018). Microbial tryptophan catabolites in health and disease. Nat. Commun..

[46] Louis P., Duncan S.H., McCrae S.I., Millar J., Jackson M.S., Flint H.J. (2004). Restricted Distribution of the Butyrate Kinase Pathway among Butyrate-Producing Bacteria from the Human Colon. J. Bacteriol..

[47] Smith P.M., Howitt M.R., Panikov N., Michaud M., Gallini C.A., Bohlooly-Y M., Glickman J.N., Garrett W.S. (2013). The Microbial Metabolites, Short-Chain Fatty Acids, Regulate Colonic Treg Cell Homeostasis. Science.

[48] Wrzosek L., Miquel S., Noordine M.-L., Bouet S., Chevalier-Curt M.J., Robert V., Philippe C., Bridonneau C., Cherbuy C., Robbe-Masselot C. (2013). Bacteroides thetaiotaomicron and Faecalibacterium prausnitzii influence the production of mucus glycans and the development of goblet cells in the colonic epithelium of a gnotobiotic model rodent. BMC Biol..

[49] Yang S.Y., Zheng Y., Huang Z., Wang X.M., Yang H. (2016). Lactococcus nasutitermitis sp. nov. isolated from a termite gut. Int. J. Syst. Evol. Microbiol..

[50] Wang J., Ji H., Wang S., Liu H., Zhang W., Zhang D., Wang Y. (2018). Probiotic Lactobacillus plantarum promotes intestinal barrier function by strengthening the epithelium and modulating gut microbiota. Front. Microbiol..

[51] Schwiertz A., Hold G., Duncan S.H., Gruhl B., Collins M.D., Lawson P.A., Flint H.J., Blaut M. (2002). Anaerostipes caccae gen. nov., sp. nov., a New Saccharolytic, Acetate-utilising, Butyrate-producing Bacterium from Human Faeces. Syst. Appl. Microbiol..

[52] Delcour A.H. (2009). Outer membrane permeability and antibiotic resistance. Biochim. Biophys. Acta (BBA)-Proteins Proteom..

[53] Vatanen T., Kostic A.D., D’Hennezel E., Siljander H., Franzosa E.A., Yassour M., Kolde R., Vlamakis H., Arthur T.D., Hämäläinen A.-M. (2016). Variation in Microbiome LPS Immunogenicity Contributes to Autoimmunity in Humans. Cell.

[54] Metges C.C. (2000). Contribution of microbial amino acids to amino acid homeostasis of the host. J. Nutr..

[55] Tomé D., Bos C. (2007). Lysine requirement through the human life cycle. J. Nutr..

[56] Wu G. (2009). Amino acids: Metabolism, functions, and nutrition. Amino Acids.

[57] Kumar M., Ji B., Babaei P., Das P., Lappa D., Ramakrishnan G., Fox T.E., Haque R., Petri W.A., Bäckhed F. (2018). Gut microbiota dysbiosis is associated with malnutrition and reduced plasma amino acid levels: Lessons from genome-scale metabolic modeling. Metab. Eng..

