# Supplementary material for: Prophylactic Treatment of Undernourished Mice with Cotrimoxazole Induces a Different Profile of Dysbiosis with Functional Metabolic Alterations
Source: Cells. 2022 Jul 23;11(15):2278. doi: 10.3390/cells11152278 (PMC9331864; doi:10.3390/cells11152278)
Supplement: Supplementary file 1 [file cells-11-02278-s001.zip › cells-1683431-supplementary.pdf]

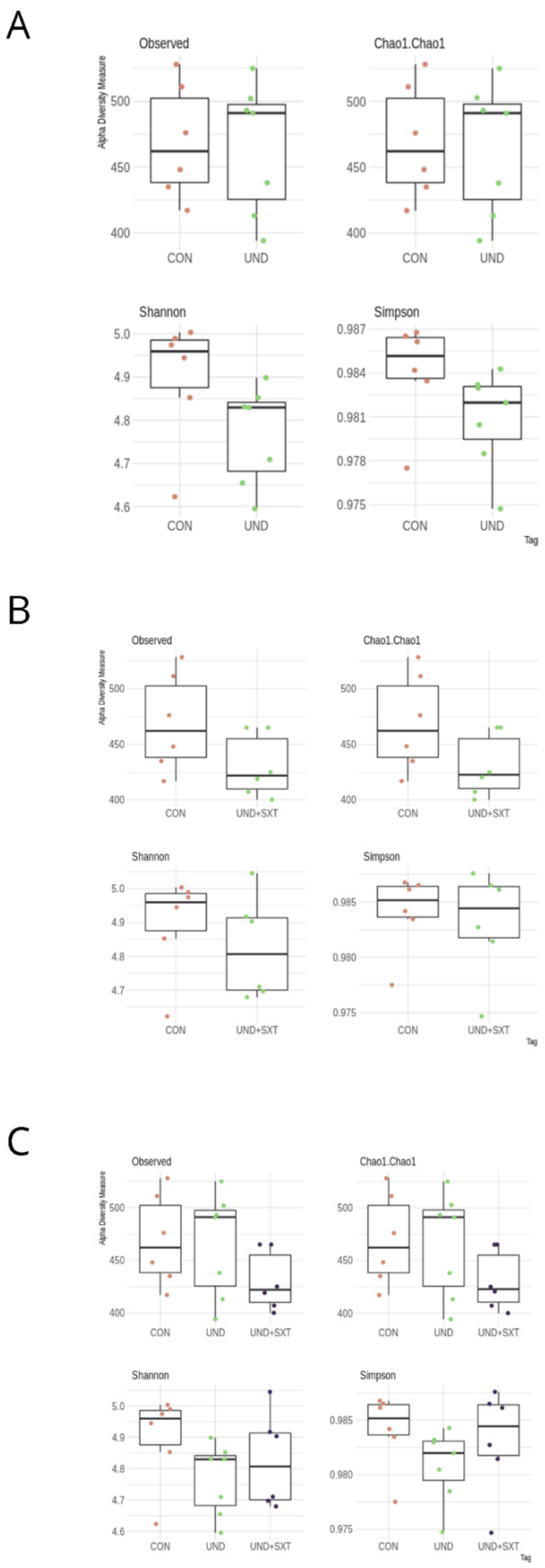

Figure S1. Richness (Observed and Chao1) and diversity (Shannon and Simpson) indexes for the gut microbiota. (a) Control (CON) and undernourished (UND) C57BL/6 mice. (b) Control (CON) and cotrimoxazole-treated undernourished (UND-SXT) C57BL/6 mice. (c) Control (CON), undernourished (UND), and cotrimoxazole-treated undernourished (UND-SXT) C57BL/6 mice.

Table S1. ANCOM-BC analysis showing W test statistic (mean difference/SE), p-value and FRD p-value for differently significant genus in the gut microbiota of CON (n = 6), UND (n = 7), and UND+SXT (n = 6) C57BL/6 mice. * Increased in UND+SXT mice when compared to CON and UND mice; ** reduced in UND+SXT mice when compared to CON and UND mice.

Family	Genus	W	p-value	FDR p-value
S24-7	Unclassified	67.039100	0.0000000	0.0000000
Enterobacteriaceae	*Klebsiella*	8.339968	0.0000000	0.0000000
Alcaligenaceae	*Sutterella***	45.898923	0.0000000	0.0000000
Porphyromonadaceae	*Parabacteroides***	16.378670	0.0005552	0.0030702
Caulobacteraceae	Unclassified	20.140173	0.0000847	0.0005601
F16	Unclassified	16.822358	0.0004447	0.0025546
Enterobacteriaceae	Unclassified	21.668384	0.0000394	0.0002756
Lachnospiraceae	*Coprococcus*	10.504023	0.0104739	0.0398010
Ruminococcaceae	*Faecalibacterium*	25.202518	0.0000067	0.0000780
Turicibacteraceae	*Turicibacter*	1.225840	0.0000000	0.0000000
Streptococcaceae	*Lactococcus***	1.612983	0.0000000	0.0000000
Pasteurellaceae	*Haemophilus***	10.896281	0.0000000	0.0000000
Clostridiaceae	*Candidatus Arthromitus***	13.439776	0.0024133	0.0114634
Clostridiaceae	Unclassified**	45.393331	0.0000000	0.0000000
Ruminococcaceae	*Anaerotruncus**	3.069544	0.0000000	0.0000000
Streptococcaceae	*Streptococcus***	25.239520	0.0000066	0.0000780
Veillonellaceae	*Veillonella***	25.138432	0.0000070	0.0000780
[Odoribacteraceae]	*Odoribacter***	14.062713	0.0017675	0.0085045
Campylobacteraceae	*Campylobacter*	24.745761	0.0000085	0.0000780
Lachnospiraceae	*Anaerostipes***	5.570561	0.0000000	0.0000000
Coriobacteriaceae	*Adlercreutzia***	16.787884	0.0000000	0.0000000
Sphingomonadaceae	*Sphingomonas*	10.801724	0.0090254	0.0355734
Clostridiaceae	*Clostridium***	53.433144	0.0000000	0.0000000
Lachnospiraceae	*Lachnospira*	25.166225	0.0000069	0.0000780
